# Evaluation of the epidemiological situation of intestinal schistosomiasis using the POC-CCA parasite antigen test and the Kato-Katz egg count test in school-age children in endemic villages in western Côte d’Ivoire[Fn FN1]

**DOI:** 10.1051/parasite/2024049

**Published:** 2024-10-29

**Authors:** Edwige A. Sokouri, Bernardin Ahouty, Innocent A. Abé, Flora G.D. Yao, Thomas K. Konan, Oscar A. Nyangiri, Annette MacLeod, Enock Matovu, Harry Noyes, Mathurin Koffi

**Affiliations:** 1 Laboratoire de Biodiversité et Gestion des Ecosystèmes Tropicaux, Unité de Recherche en Génétique et Epidémiologie Moléculaire, UFR Environnement, Université Jean Lorougnon Guédé Daloa Côte d’Ivoire; 2 College of Veterinary Medicine, Animal Resources and Biosecurity, Makerere University Kampala Uganda; 3 College of Medical, Veterinary and Life Sciences Institute of Biodiversity, Animal Health and Comparative Medicine, University of Glasgow Glasgow UK; 4 Centre for Genomic Research, University of Liverpool Liverpool UK

**Keywords:** Intestinal schistosomiasis, Prevalence, Intensity, Risk factors, Western Côte d’Ivoire, KK, POC-CCA

## Abstract

Schistosomiasis is an endemic disease in Côte d’Ivoire. We compared the conventional Kato Katz (KK) test and a more sensitive but rarely used method, the point-of-care circulating cathodic antigen (POC-CCA), in order to contribute to the development of a more appropriate strategy for the control and elimination of intestinal schistosomiasis in western Côte d’Ivoire. A cross-sectional epidemiological survey was conducted in eight elementary schools in the Guémon and Cavally regions from February to December 2020. Selected schoolchildren provided stool and urine samples to detect the presence of *Schistosoma mansoni* eggs and parasite antigen using the KK and POC-CCA tests, respectively. A total of 554 schoolchildren were included in the study. The overall prevalence of intestinal schistosomiasis was 10% and 67% for KK and POC-CCA, respectively. The POC-CCA detected an infection rate of 100%, while the KK yielded a rate of 42%. In schools, prevalence ranged from 27 to 100% with POC-CCA and from 0 to 42% with KK. Swimming, fishing, washing clothes, and dishwashing were significantly associated with the onset of infection and high intensities. The epidemiological risk factors for intestinal schistosomiasis updated here using KK and POC-CCA diagnostic methods showed that prevalence was much higher than previously estimated using the KK. The POC-CCA is more sensitive and ways should be considered to improve its specificity in order to improve the diagnosis.

## Abstract (fr)

La schistosomiase est une maladie endémique en Côte d’Ivoire. Nous avons comparé le test conventionnel Kato Katz (KK) et une méthode plus sensible mais rarement utilisée, l’antigène cathodique circulant au point d’intervention (POC-CCA), afin de contribuer au développement d’une stratégie plus appropriée pour le contrôle et l'élimination de la schistosomiase intestinale dans l’ouest de la Côte d’Ivoire. Une enquête épidémiologique transversale a été menée dans huit écoles élémentaires des régions de Guémon et Cavally de février à décembre 2020. Des écoliers sélectionnés ont fourni des échantillons de selles et d’urine pour détecter la présence d’œufs de *Schistosoma mansoni* et d’antigène parasitaire à l’aide respectivement des tests KK et POC-CCA. Au total, 554 écoliers ont été inclus dans cette étude. La prévalence globale de la schistosomiase intestinale était respectivement de 10% et 67% pour KK et POC-CCA. Le POC-CCA a détecté un taux d’infection de 100%, tandis que le KK a donné un taux de 42%. Dans les écoles, la prévalence variait de 27 à 100% avec POC-CCA et de 0 à 42% avec KK. La natation, la pêche, la lessive et la vaisselle étaient associées de manière significative à l’apparition de l’infection et à des intensités élevées. La mise à jour ici des facteurs de risque épidémiologiques de la schistosomiase intestinale à l’aide des méthodes de diagnostic KK et POC-CCA a montré que la prévalence était beaucoup plus élevée que celle estimée précédemment à l’aide du KK. Le POC-CCA est plus sensible et des moyens devraient être envisagés pour améliorer sa spécificité afin de rendre un meilleur diagnostic.

## Introduction

Human schistosomiasis or bilharzia is a waterborne disease caused by trematodes of the genus *Schistosoma* [20, 32]. Human infection occurs in water via freshwater snails, which serve as intermediate hosts and vectors [2, 20]. Classified as a neglected tropical disease, human bilharzia is considered the most important helminthic disease of humanity in terms of morbidity and mortality [63]. It is endemic in low-income regions, particularly in sub-Saharan Africa. In this part of Africa, two main forms of the disease have been identified: the intestinal form caused by *Schistosoma mansoni* and the urinary form involving *Schistosoma haematobium*. Every year, more than 112 million people are affected by bilharzias and between 150,000 and 200,000 deaths are estimated [39, 45, 58]. *Schistosoma mansoni* is the most common parasite of human schistosomiasis [32, 65]. The disease is particularly prevalent in school-age children [5, 35] who contract the parasite during domestic activities such as laundry, dishwashing and water collection, as well as during recreational activities such as swimming and playing in rivers infected with the parasite.

Several environmental factors have been examined in the epidemiology of schistosomiasis, including temperature, rainfall, altitude, soil, vegetation and intermediate host ecology [15, 36, 37]. All these factors, combined with poor hygiene conditions, favour the onset and spread of the disease.

In Côte d’Ivoire, intestinal schistosomiasis is endemic in the south, centre and west of the country [4, 6]. In the western region, prevalence can reach 100% in certain localities [7], despite the mass treatments administered each year [8]. In Côte d’Ivoire, diagnosis of cases and determination of the intensity of schistosomiasis infection have almost always been carried out using the Kato Katz (KK) technique, which has high specificity but low sensitivity [3, 7, 8, 53]. On rare occasions, the point-of-care circulating cathodic antigen (POC-CCA) technique has also been used. POC-CCA is a urine test for active schistosome worm infections, providing a semi-quantitative rather than quantitative estimate of the intensity of infection, but is much more sensitive than the KK test and may lead to an overestimate of the true prevalence due to false positives [10, 33]. The characteristics of these two tests highlight the urgent need to further compare and understand the variability between POC-CCA and KK results. Importantly, the use of sensitive and specific diagnostic tools is essential for accurate surveillance and to support progress towards the elimination of schistosomiasis. To establish an effective diagnosis, diagnostic methods must be accurate, simple and affordable for all diagnostic facilities and provide results within a short time in clinical services [64]. To achieve this, we complemented the existing KK technique with a measurement of the intensity of infection (POC-CCA) using an electronic reader to accurately determine the intensity of the band in the POC-CCA test and to provide an appropriate quantitative test [48]. This provides more accurate and consistent results on infection status and represents a more appropriate strategy to control infection in the region. The aim of our study was to update the epidemiological situation of *Schistosoma mansoni* bilharzia in western Côte d’Ivoire using two different diagnostic methods. It aimed to contribute to understanding the environmental determinants and persistence of intestinal schistosomiasis in the west of the country. Our results may contribute to the development of a more appropriate strategy for infection control and elimination of the disease in western Côte d’Ivoire.

## Materials and methods

### Ethical considerations

This study is part of the TrypanoGEN+ project conducted in Uganda, Democratic Republic of Congo (DRC), Cameroon and Côte d’Ivoire for intestinal bilharzia. The study protocol was approved by the National Ethics Committee for Life Sciences and Health (CNESVS) under approval number N/Ref: 040-19/MSHP/CNESVS-kp. The study also received authorisation from regional, departmental, village health and education authorities. Parents of pupils were informed of the purpose of the study in their own language. Parents agreed to their child’s participation in the study and signed an informed consent form, in addition to oral assent obtained from children. When the analyses (KK or POC-CCA) were positive, the participants were treated with praziquantel.

### Study area

**Figure 1:**
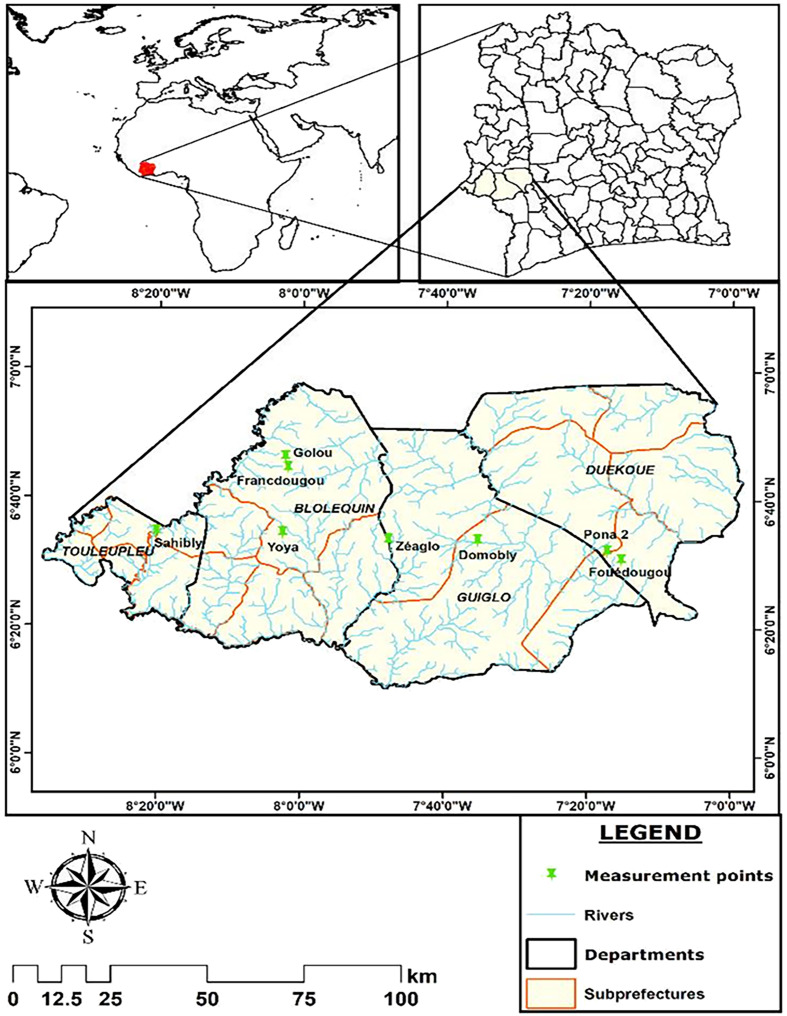
Map of the regions of Guémon and Cavally indicating the schools of the surveyed localities. map obtained from: https://www.qgis.org/fr/site/forusers/download.html with layers downloaded via the link: https://www.diva-gis.org/ (consulted 18/03/2022).

This study took place in the western part of Côte d’Ivoire in two regions of the Mountain District, which is located between latitudes 06°10′N and 06°80′N and longitudes 8°39′W and 7°0′W (Fig. 1). These regions are Guémon (Duékoué) and Cavally (Guiglo, Bloléquin and Toulepleu) [8]. This is a forested and mountainous area watered by the Sassandra and Cavally rivers. This area was chosen because of high prevalence of *S. mansoni* reported in previous studies [4, 8]. The sites of Pona (PON), Fouédougou (FOU) (Duékoué), Domobly (DOM) (Guiglo), Zéaglo (ZEA), Francdougou (FRA), Yoya (YOY), Golou (GOL) (Bloléquin) and Sahibly (SAH) (Toulepleu) were selected following an environmental and malacological survey that showed that both rivers had many riparian communities and were also infested with *Biomphalaria pfeifferi*, the only intermediate host of *S. mansoni* recorded in Côte d’Ivoire.

### Study design and population

This was a cross-sectional study carried out between February and December 2020 in a school setting. The participants were schoolchildren selected by random sampling from eight schools in two regions of western Côte d’Ivoire.

### Inclusion and exclusion criteria

The villages were selected on the basis of the presence of the mollusc *Biomphalaria pfeifferi*, the only known intermediate host of *Schistosoma mansoni* in Côte d’Ivoire. The study included schoolchildren who had been resident for at least two years and were attending school in the selected village, and whose parents or guardians had given their informed consent. The schoolchildren also gave their assent. Participants had to have at least one biological parent and one first-degree relative available and be between 5 and 14 years of age. Children with a history of treatment with praziquantel in the last six months and those under 5 and over 14 years of age were excluded.

### Questionnaires to school children

The school children and their parents were informed about the purpose of the study, how they should collect their samples, and how the samples would be processed by the research team. When parents permitted their children to participate in the study, a questionnaire was used to collect socio-demographics information such as age, gender and activities related to contact with freshwater. This questionnaire also included information about praziquantel intake.

### Detection of *Schistosoma mansoni* eggs by the Kato-Katz test

Each school child included in this study provided a stool sample of approximately 20 g. These samples were processed the same day by the Kato-Katz technique to test for *S. mansoni* eggs according to the WHO standard procedure [38]. Each sample was prepared using a sieve and a smear 41.7 mg thick was taken on two slides. The slides were read by two different experienced technicians using a microscope. For quality control purposes, 10% of the slides were randomly drawn and then re-examined. The sample was considered positive to the Kato-Katz test when an *S. mansoni* egg was observed. Then, *S. mansoni* eggs were counted per slide, multiplied by 24, and the arithmetic means of the two slides were expressed as the number of eggs per gram (EPG) of stool [38, 66, 67]. The intensity of infection was classified as low (1–99 EPG), moderate (100–399 EPG) and high (≥400 EPG) [14, 47].

### Detection of *Schistosoma mansoni* circulating cathode antigen by POC-CCA

The POC-CCA technique is a rapid diagnostic serological method implemented to detect the adult egg antigen of *S. mansoni* [16, 29]. The POC-CCA test was performed according to the manufacturer’s instructions (ICT Diagnostics, Cape Town, South Africa). Thus, schoolchildren provided approximately 50 mL of urine in collection boxes on the spot. The test was conducted in two steps.

The first step was to detect the presence or absence of CCA and to determine the semi-quantitative intensity (visual detection based on the intensity of staining of the test line) of the infection in the urine sample.

For this purpose, two drops of urine from each individual were inoculated into the cassette well. After 20 min of complete absorption and incubation, the reading was taken and the semi-quantitative result was given based on the absence or presence and intensity of staining of the test line. Results were scored as follows: Negative (absence of test line staining), Trace, +1, +2, +3 (depending on the intensity of test line staining) [48, 54, 62].

The second step consisted of the quantification of the CCA.

After annotation of the semi-quantitative result, the cassette was introduced into an ESEQuant LR3 reader (QIAGEN, Hilden, Germany) previously calibrated with samples containing different concentrations of CCA provided by the University of Leiden, The Netherlands implemented in the LF Studio application. The amount of antigen (CCA) is correlated to the intensity of the staining of the test strip, which is expressed in millivolts for each sample. This millivolt value is therefore considered a relative measurement of the amount of CCA present in the urine sample.

### Statistical analysis

All data collected were entered into Excel and then Rstudio was used to import the data into R software, version 4.3.3 [60] for analysis. To identify the factors responsible for the maintenance of intestinal schistosomiasis in the study area, independent qualitative variables were defined. These were socio-demographic factors (schools, departments, regions, sex, age) and behavioural factors (swimming, washing, fishing). The quantity of eggs and the quantity of CCA were considered to be dependent quantitative variables.

Simple logistic regression was used to test for associations between the infection status variable and potential factors. The association of the factors with the intensity of infection was performed using Negative Binomial Generalised Linear Models (NB-GLM) and Negative Binomial Generalised Linear Mixed Models (NB-GLMM) for POC-CCA data and Negative Binomial Logit Hurdle (NBLH) for Kato-Katz data for the bivariate analysis. Partial missing data were removed for each variable. School or region were modeled as random factors in NB-GLMM. Values of *p* < 0.05 were considered significant.

To compare the performance of the POC-CCA and Kato Katz tests, only samples with data from both tests were considered. Sensitivity, specificity, positive predictive value (PPV), negative predictive value (NPV), accuracy and Kappa value were the parameters considered in this study.

## Results

### Prevalence of intestinal schistosomiasis

A total of 610 registered schoolchildren were potentially eligible. Of these, 56 either did not provided a sample (stool or urine) or did not give consent. Thus, 554 from the eight schools were included in the study. However, among these pupils, there was some missing data in terms of gender, age and Kato Katz results. As a result, 550 pupils were considered for the calculation of the sex ratio (265 girls and 285 boys). The ratio of boys to girls was 1.07. The mean age of the children sampled was 9.35 ± 2.07 years.

**Figure 2:**
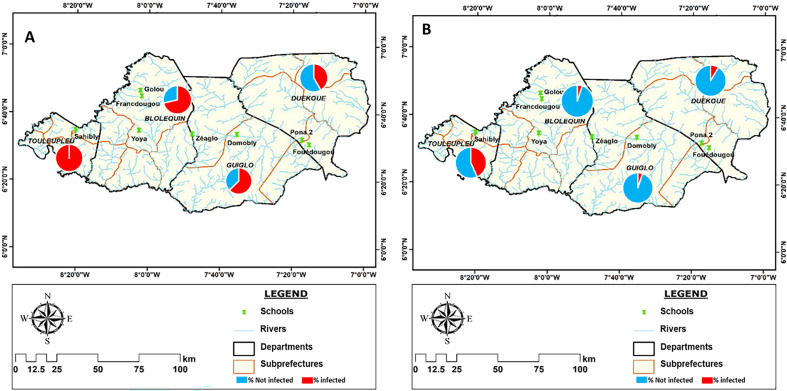
Distribution of prevalence per departments according with baseline layers obtained from: https://www.diva-gis.org/. A: Prevalence distribution according to the POC-CCA test, B: Prevalence distribution according to Kato-Katz test.

**Figure 3:**
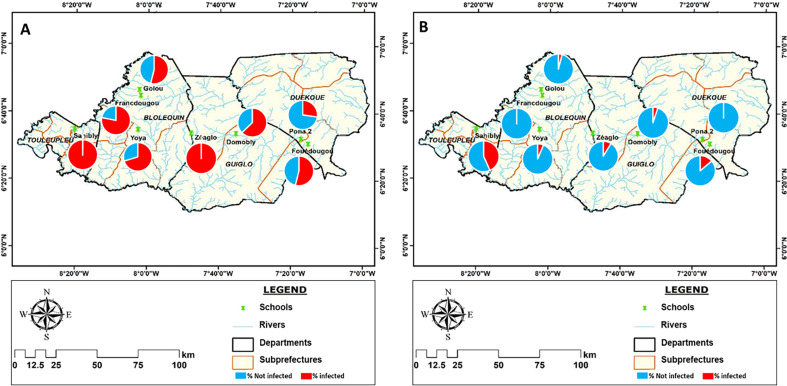
Distribution of prevalence per school according with baseline layers obtained with baseline layers obtained from: https://www.diva-gis.org/. A: Prevalence distribution according to the POC-CCA test, B: Prevalence distribution according to Kato-Katz test.

The overall prevalence of *S. mansoni* detected by the diagnostic tests was 67% for the POC-CCA and 10% for the Kato-Katz test (KK) (Table 1). The population of the Cavally region (74% and 10% for POC-CCA and KK, respectively) was more infected with intestinal schistosomiasis than that of Guémon (42% and 9%, respectively) (Table 1). A total of 87% of individuals positive by POC-CCA and 83% for KK were detected in Cavally. The highest infection rates detected by POC-CCA were obtained in Toulepleu (100%) and Bloléquin (71%) in the department of Cavally. Toulepleu had the highest rate of *S. mansoni* egg excretors (42%) (Table 1 and Fig. 2). In the schools, prevalence ranged from 27% to 100% with POC-CCA and from 0% to 42% with KK. However, in Francdougou in Cavally and Pona in the Guémon region, no schoolchildren excreted eggs, although the antigen test (POC-CCA) indicated infections. The most affected schools were Sahibly and Zéaglo, in the departments of Toulepleu and Guiglo, respectively with a 100% infection rate by POC-CCA (Fig. 3). The overall prevalence among boys was 73% by POC-CCA and 13% by KK compared to 60% with POC-CCA and 6% with KK among girls. The age group 12–14 years recorded the highest prevalence (84% with POC-CCA and 27% with KK) of infection compared with the others (Table 1).

**Table 1:** Prevalence of intestinal schistosomiasis in relation to sites and demographic characteristics.

Variable	No. tested	Positive POC-CCA (%)	No. Tested	Positive KK (%)
Socio-demographic factors				
Fouédougou	65	35 (54)	65	9 (14)
Pona 2	55	15 (27)	40	0 (0)
Domobly	59	37 (63)	59	3 (5)
Francdougou	50	39 (78)	50	0 (0)
Golou	100	54 (54)	100	4 (4)
Sahibly	60	60 (100)	60	26 (43)
Yoya	111	78 (70)	111	7 (6)
Zéaglo	54	54 (100)	57	5 (9)
Total	554	372 (67)	542	54 (10)
Departments				
Bloléquin	315	225 (71)	318	16 (5)
Duékoué	120	50 (47)	105	9 (9)
Guiglo	59	37 (63)	59	3 (5)
Toulepleu	60	60 (100)	60	26 (43)
Total	554	372 (67)	542	54 (10)
Region				
Guémon	120	50 (42)	105	9 (9)
Cavally	434	322 (74)	437	45 (10)
Total	554	372 (67)	542	54 (10)
Gender				
Male	285	209 (73)	277	37 (13)
Female	265	160 (60)	260	17 (07)
Total	550		537	
Age (year)				
05–07	118	74 (63)	109	6 (6)
08–09	127	89 (70)	114	13 (11)
10–11	203	143 (70)	184	21 (11)
12–14	70	59 (84)	57	14 (27)
Total	518		464	

### Spatial distribution of prevalence

Figures 2 and 3 show the distribution of intestinal schistosomiasis infection in the surveyed departments and schools. The results showed a significant variation in prevalence in the schools, departments and regions of interest. The POC-CCA test showed cases of schistosomiasis in 100% of surveyed schools and KK showed cases of intestinal schistosomiasis in 75% of schools. Also, cases of infection were recorded in 100% of the departments and regions visited, based on the results of both tests. Therefore, prevalence ranged from 27% to 100% for POC-CCA and from 0% to 43% for KK in schools. At the departmental level, the prevalence was between 47% and 100% for the POC-CCA and between 5% and 43% for KK (Table 1). Only one school (Pona) with 12.5% prevalence and only one department (Duékoué) with 25% prevalence had a prevalence of less than 50% according to the POC-CCA test; however, all schools and departments had a prevalence less than 50% for KK.

### Risk factors associated with infection status

Logistic regression analysis was conducted to identify factors likely to influence the occurrence of the disease. The risk of being infected was higher for students in Sahibly school (*p* < 0.001) relative to Domobly students. Bloléquin (*p* < 0.001) and Guiglo (*p* = 0.006) departments had significantly higher prevalence compared to Duékoué, and the Cavally region (*p* < 0.001) compared to Guémon. The students in the Pona school had a significantly lower risk of being infected (*p* < 0.001). The results of this analysis also revealed that the infection rate in boys was 1.9 times higher than in girls by KK (odds ratio = 2.15, *p* = 0.019) and 1.2 times higher by POC-CCA (*p* = 0.007) (Tables 1 and 2). The 12–14 years age group was three to four times more likely to be KK positive than the 5–7 years age group (*p* = 0.004), but only 33% more likely to be infected by POC-CCA (*p* = 0.002). The data from the KK test indicated that activities in contact with water such as swimming (*p* = 0.005), fishing (*p* < 0.001) and washing clothes (*p* = 0.002) were significantly associated with the probability of infection by *S. mansoni* (Table 2). However, when the POC-CCA data were analysed with all outcomes without taking into account the intensity level of the responses, no water-related activities were significant. Additionally, when the analyses were performed considering as positive the samples with a higher intensity based on the visual result of the POC-CCA cassette (+3), swimming (*p* = 0.013), fishing (*p* = 0.001) and laundry (*p* < 0.001) were significantly associated with the probability of *S. mansoni* infection (Table 2). Water contact activities such as fishing (*p* < 0.001) and washing clothes (*p* = 0.007) were significantly associated with the probability of *S. mansoni* infection (Table 2).

**Table 2:** Logistic regression analysis of predictors of intestinal schistosomiasis infection prevalence in the study population.

Factor	POC-CCA			KK		
	OR	95%CI	*p*-value	OR	95%CI	*p*-value
Socio-demographic factors						
School						
Domobly	–	–	–	–	–	–
Francdougou	2.11	1–4.63	0.057	1.61e^−07^	2.56e^−136^–5.52e^+07^	0.98
Golou	0.70	0.38–1.25	0.23	0.78	0.19–3.51	0.727
Sahibly	6.88^+07^	4.23e^+129^–7.33e^+143^	0.98	13.33	4.64–51.33	<0.001*
Yoya	1.40	0.77–2.55	0.26	1.26	0.0.37–5.21	0.726
Zéaglo	6.88e^+08^	5.83e^+135^–1.67e^+151^	0.99	1.80	0.47–7.87	0.40
Pona	0.22	0.11–0.45	<0.001*	1.61e^−07^	1.61e^−151^–2.87e^+09^	0.99
Fouédougou	0.69	0.36–1.32	0.27	3	0.93–12.20	0.085
Departments						
Duékoué	–	–	–	–	–	–
Bloléquin	3.50	2.32–5.32	<0.001*	0.21	0.07–0.62	0.004*
Guiglo	2.35	1.29–2.36	0.006*	0.12	0.01–0.75	0.059
Toulepleu	5.96 e^+07^	0.96–8.96 e^+83^	0.97	3.61	1.43–10.11	0.010*
Region Cavally	4.02	2.65–6.17	<0.001*	1.19	0.59–2.69	0.64
Sex male	1.82	1.18–2.82	0.007*	2.15	1.15–4.20	0.019*
Age	1.17	1.07–1.29	0.001*	1.23	1.07–1.43	0.005*
05–07	–	–	–	–	–	–
08–09	1.39	0.82–2.38	0.223	1.90	0.71–5.61	0.22
10–11	1.42	0.87–2.29	0.154	2.07	0.86–5.79	0.13
12–14	3.19	1.56–6.99	0.002*	4.46	1.69–13.18	0.004*
Behavioural factors						
Swimming	1.06	0.69–1.63	0.776	2.61	1.37–5.26	0.005*
Swimming^+3^	1.87	1.15–3.09	0.013*			
Fishing	1.39	0.88–2.22	0.162	2.95	1.61–5.51	<0.001*
Fishing^+3^	2.29	1.42–3.70	0.001*			
Washing clothes	1.38	0.88–2.23	0.168	2.64	1.44–4.90	0.002*
Washing clothes^+3^	2.76	1.67–4.61	<0.001*			
Washing dishes	0.94	0.56–1.64	0.775	1.48	0.71–2.92	0.272
Washing dishes^+3^	2.06	1.15–3.61	0.013*			

^+3^ = regression performed with high intensity based on visual result of the POC-CCA cassette (+3), KK = Kato Katz, OR = Odds Ratio, POC-CCA = Point of Care Circulating Cathodic Antigen.

*Significant analysis.

### Intensity of intestinal schistosomiasis

**Figure 4:**
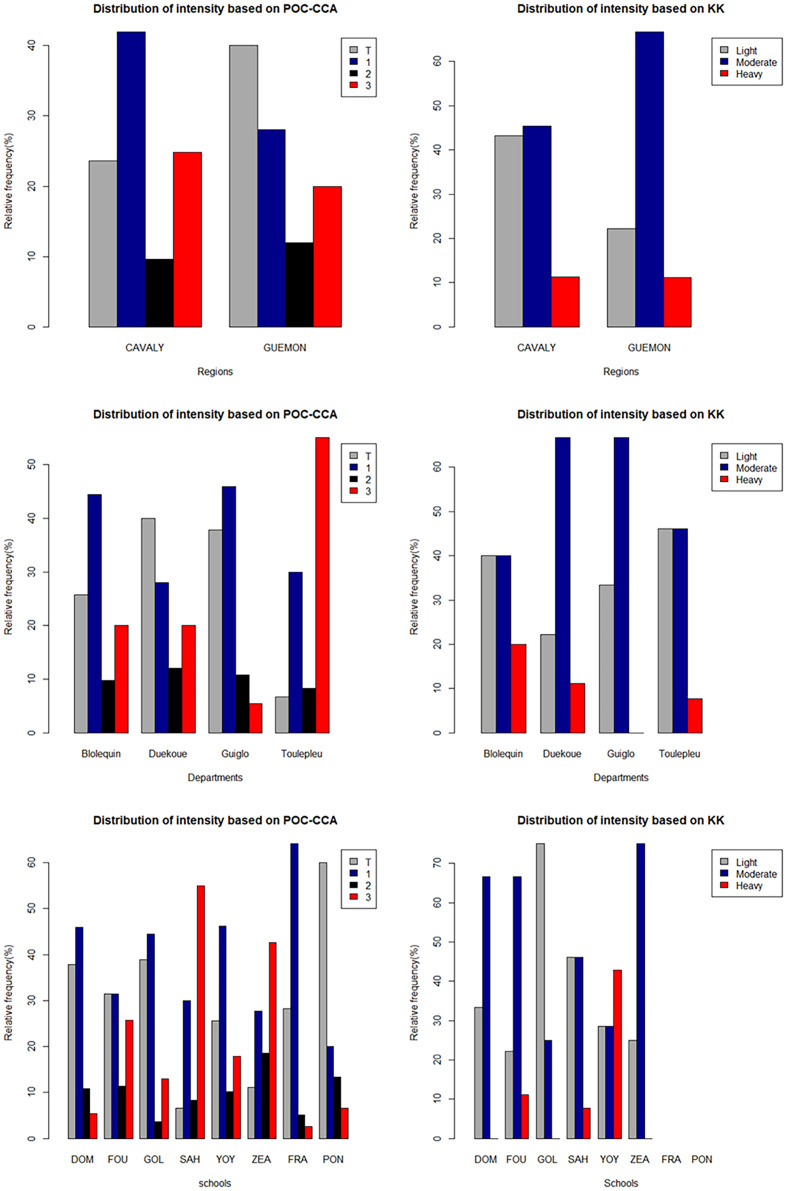
Semi-quantitative POC-CCA and Kato-Katz intensity of infection results per school, departments and region. DOM = Domobly, FOU = Fouédougou, FRA = Francdougou, GOL = Golou, PON = Pona 2, SAH = Sahibly, YOY = Yoya, ZEA = Zéaglo.

The average number of *S. mansoni* eggs detected in the stool of all positive individuals was 199 EPG. Among the positive individuals, 39% had a low intensity of infection (1–99 EPG), 50% a moderate intensity of infection (100–399 EPG) and 11% a high intensity of infection (≥400 EPG), according to the WHO classification (Fig. 4). In all, 83% of severe infections were recorded in the Cavally region (Fig. 4). The semi-quantitative result of the POC-CCA in the overall population was 96 (26%) for Trace, 149 (40%) for +1, 37 (10%) for +2 and 90 (24%) for +3 (Fig. 4). The average amount of CCA in the positives was 257 mV.

High levels of CCA (+3) (visual result of the POC-CCA test or semi-quantitative diagnosis) were mainly recorded in the Cavally region (90%), in the departments of Bloléquin (51%) and Toulepleu (37%) and the schools of Sahibly (37%) and Zéaglo (25%). Of the 6 heavy infections detected by KK, 5 were from the Cavally region, 3 from the school of Yoya in the department of Bloléquin and 2 from the school of Sahibly in Toulepleu department. However, in Zéaglo, high levels of circulating antigen (CCA) were detected, but no heavy egg burdens in the infected cases (Fig. 4).

### Spatial distribution of infection intensity

**Figure 5:**
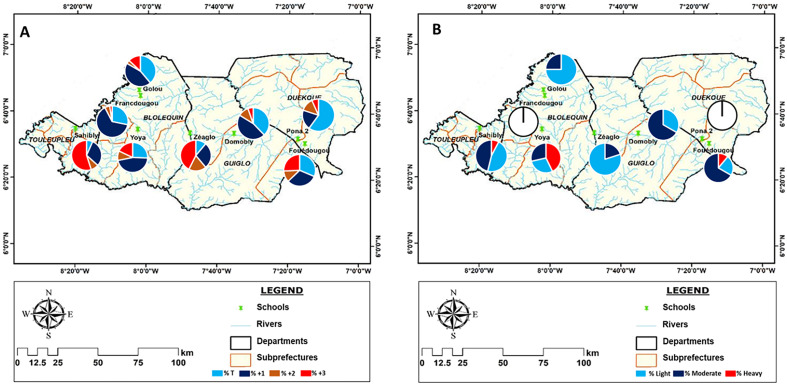
Distribution of infection intensities per school of positive schoolchildren with baseline layers obtained from: https://www.diva-gis.org/. A: intensities distribution according to the POC-CCA test, B: Intensities distribution according to Kato Katz test.

**Figure 6:**
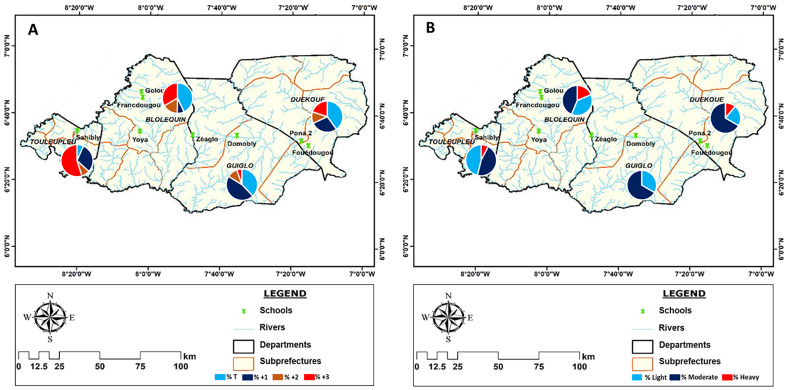
Distribution of infection intensities per department of positive schoolchildren with baseline layers obtained from: https://www.diva-gis.org/. A: intensities distribution according to the POC-CCA test, B: Intensities distribution according to Kato-Katz test.

The spatial distribution of infection intensity based on the visual result of the POC-CCA and the KK is shown in Figures 5 and 6. The different levels of infection intensity were heterogeneously distributed over the surveyed sites. High infection intensities (+3 for POC-CCA) were found for all schools, departments and regions, but with different proportions. They ranged from 2% to 55% of the positive cases. In Sahibly school and Toulepleu department, infection level +3 accounted for over half (55%) of the positive cases. On the other hand, in the schools of Domobly, Francdougou, Golou and Pona, low levels of infection (Trace and +1) represented more than 70% of the proportion of positive results. The same is true for the departments of Duékoué and Guiglo. High intensities of infection based on KK were found in 37.5% of schools and 75% of departments. High intensities ranged from 0% to 42% in schools and from 0% to 18.75% in departments, with Yoya and Bloléquin having the highest percentages of high intensities. Low intensities were found in all schools and departments, except for the schools in Pona and Francdougou, where no cases were detected. Their proportion varied between 0% and 75% in the schools and between 7% and 18.75% in the departments (Fig. 6).

### Risk factors associated with the intensity of infection

The GLM between the POC-CCA results and the potential risk factors swimming, fishing, laundry and washing up showed non-significant associations (Table 3). A GLM was performed with the high infections (+3) and this showed significant associations with swimming (*p* = 0.013), fishing (*p* < 0.001), laundry (*p* < 0.001) and dish washing (*p* = 0.013).

**Table 3:** Bivariate analysis based on NB-GLM and NBLH with random factor exclusion.

Variable	POC-CCA			KK		
	OR	95%CI	*p*-value	OR	95%CI	*p*-value
Socio-demographic factors						
Schools						
Domobly	–	–	–	–	–	
Francdougou	0.58	0.25–1.71	0.848	1.61e^−07^	0.00–Inf	0.992
Golou	0.91	0.62–1.34	0.647	0.78	0.17–3.60	0.748
Sahibly	4.87	3.17–7.49	<0.001*	14.27	4.01–50.77	<0.001*
Yoya	1.78	1.19–2.65	0.004*	1.45	0.35–6.07	0.607
Zéaglo	3.79	2.27–6.51	<0.001*	2.07	0.39–10.96	0.390
Pona	0.58	0.25–1.71	0.262	1.61e^−07^	0.00–Inf	1.000
Fouédougou	2.29	1.40–3.82	0.001*	4.67	1.12–19.43	0.034*
Departments						
Duékoué	–	–	–	–	–	–
Bloléquin	0.76	0.49–1.12	0.190	0.26	0.10–0.70	0.007*
Guiglo	0.50	0.30– 0.82	0.006*	0.27	0.06–1.10	0.068
Toulepleu	2.43	1.47–3.97	<0.001*	3.83	1.47–9.97	0.006*
Region Cavally	0.98	0.62–1.47	0.937	0.61	0.26–1.48	0.279
Sex Male	1.36	1.05–1.74	0.017*	2.23	1.18–4.23	0.014*
Age	1.14	1.07–1.22	<0.001*	1.20	1.02–1.40	0.024*
05–07	–	–	–	–	–	–
08–09	1.52	1.02–2.24	0.037*	1.73	0.59–5.09	0.317
10–11	1.48	1.018–2.11	0.035*	1.41	0.50–3.98	0.515
12–14	2.32	1.49–3.62	<0.001*	3.62	1.22–10.72	0.020*
Behavioural factors						
Swimming	1.25	0.97–1.61	0.087	2.41	1.25–4.62	0.008*
Fishing	1.43	1.10–1.87	0.008*	2.80	1.53–5.13	0.001*
Washing clothes	1.58	1.12–2.07	0.001*	2.52	1.38–4.61	0.003*
Washing dishes	1.32	0.97–1.85	0.089	1.44	0.71–2.90	0.31

*Significant analysis.

**Figure 7:**
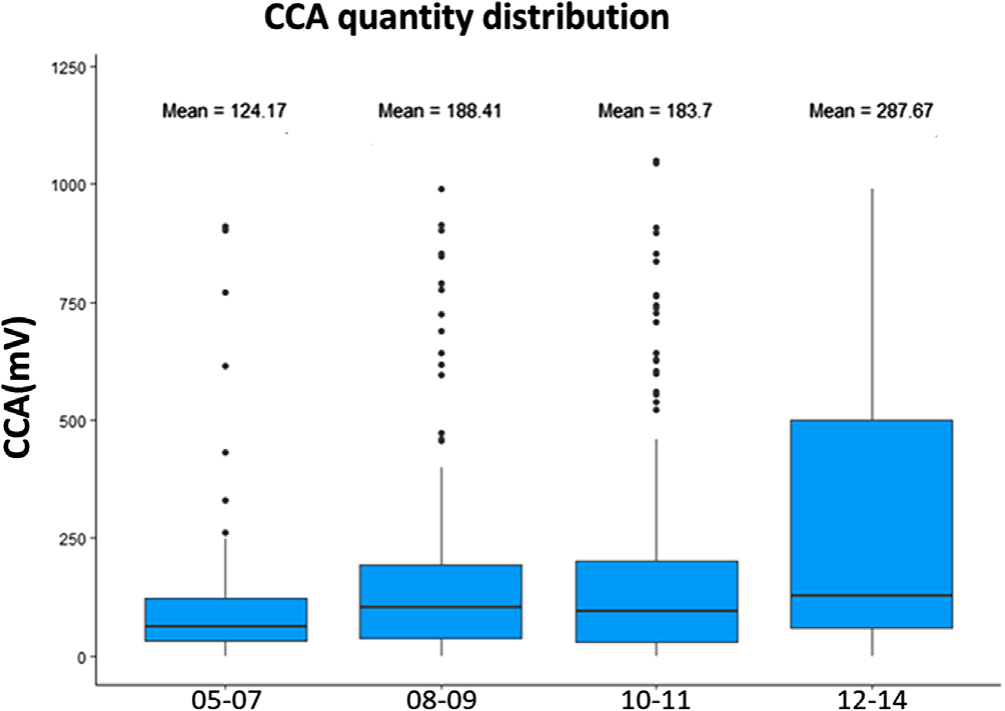
Box plot of infection intensities according to age.

The bivariate analyses using NB-GLM on the POC-CCA results and HNBL on the KK results were run with and without the effect of site (schools, departments, and regions). The effect of schools, departments and regions were correlated and therefore each of these variables was tested separately. Results are shown in Table 3. Based on both the POC-CCA and KK tests, the schools in Sahibly (*p* < 0.001 for POC-CCA and *p* < 0.001 for KK), Fouédougou (*p* = 0.001 for POC-CCA; *p* = 0.034 for KK) and the department of Toulepleu (*p* < 0.001; *p* = 0.006 respectively for POC-CCA and KK) had significantly more cases of high intensity infection than the school of Domobly or department of Duékoué respectively. On the other hand, on the basis of the POC-CCA test alone, Yoya (*p* = 0.004) and Zéaglo (*p* < 0.001) schools, in addition to the first two mentioned, were significantly more likely to have high intensity infections. The department of Bloléquin (*p* = 0.007) and Guiglo (*p* = 0.006) were negatively associated with high circulating antigen production and *S. mansoni* egg excretion relative to Duékoué. 41% of girls and 59% of boys had high intensity by POC-CCA (+3) infection (*p* = 0.017 for POC-CCA), and 33% of girls and 67% of boys had high intensity infections by KK (≥400 EPG) (*p* = 0.014). Analysis with age as the explanatory variable showed that intensity of infection increased with age (*p* < 0.001 for POC-CCA and *p* = 0.024 for KK) and that the 12–14 age group (*p* < 0.001 for POC-CCA and *p* = 0.020 KK relative to 5–7-year-olds) had the highest intensities of infection (when a second analysis was performed with all four age groups as modalities of the age variable) (Fig. 7). Children engaged in swimming (*p* = 0.008), fishing (*p* = 0.008 for POC-CCA; *p* = 0.001 for KK) and laundry (*p* = 0.001 for POC-CCA; *p* = 0.003 for KK) were more likely to have heavy infections.

When the sites were included as random factors in the NB-GLMM, gender (*p* = 0.005), age (*p* = 0.001) remained significant but the other factors became insignificant (Table 4). This is consistent with the effects of site being correlated with the environmental effects of swimming, fishing and clothes washing. Since the swimming, fisshing and clothes washing involve exposure to the infective cercariae it is likely that these are true associations that are masked by the correlated site variables when these are included in the same analysis.

**Table 4:** Negative binomial generalised linear mixed model (NB-GLMM) for intensity.

Variable	OR	95%CI	*p*-value
Socio-demographics factors			
Sex Male	1.32	1.03–1.68	**0.026**
Age			
05–07	–	–	–
08–09	1.24	0.85–1.83	0.267
10–11	1.33	0.93–1.90	0.115
12–14	2.10	1.37–3.22	**0.001**
Behavioural factors			
Swimming	1.12	0.87–1.45	0.385
Fishing	1.08	0.84–1.39	0.561
Washing clothes	1.17	0.89–1.53	0.265
Washing dish	1.32	0.96–1.81	0.088

Bold value: Significant analysis.

### Sensitivity and specificity of diagnostic tests

To determine the efficacy parameters of the two tests, the Kato Katz was used as the standard method of analysis (Table 5). The results show that no negative individual in the POC-CCA test was positive for KK.However, when the traces were considered positive (POC-CCA(t+)) 85.52% of individuals were KK-negative and when the traces were considered negative (POC-CCA(t-)) 80.59% were KK-negative. These results indicate that the POC-CCA test is highly sensitive for detecting *S. mansoni* infections (100% [93.28%, 100%]). However, when the traces were considered positive or negative, the analyses gave different results for the following parameters: specificity, NPV, precision and Kappa value. When the traces were considered POC-CCA(t+) positive, the concordance of the two tests was 42% (*k* = 0.098) with for specificity 36%. However, when the traces were considered POC-CCA(t-) negative, the agreement (59%, *k* = 0.192) and specificity seemed to improve (from 36% [31%, 40%] to 55% [50%, 59%]).

**Table 5:** Sensitivity, specificity and Kappa scores of the POC-CCA test versus Kato-Katz results.

POC-CCA(t+)	KK			POC-CCA(t−)	KK		
	Positive (%)	Negative (%)	Total		Positive (%)	Negative (%)	Total
Positive	53 (14.48)	313 (85.52)	366	Positive	53 (19.41)	220 (80.59	273
Negative	0 (0)	173 (100)	173	Negative	0 (00)	266 (100)	266
Total	53	486		Total	53	486	
Sensitivity	100 (93.28, 100)				100 (93.28, 100)		
Specificity	35.60 (31.34, 40.03)				54.73 (50.19, 59.22)		
PPV	14.48 (11.04, 18.51)				19.41 (14.89, 24.61)		
NPV	100 (97.89, 100)				100 (98.62, 100)		
Accuracy	41.93 (37.73, 46.22)				59.18 (54.90, 63.37)		
*k*	0.098 (0.07, 0.13)				0.192 (0.14, 0.24)		

### Discussion

Intestinal schistosomiasis remains a major public health problem, especially in western Côte d’Ivoire, despite the efforts made by the national programme to control this endemic, in contrast to Egypt, which has succeeded in eliminating schistosomiasis [1, 24]. The aim of the present study was to contribute to the development of a more appropriate strategy for infection control and elimination of intestinal schistosomiasis in the endemic area of western Côte d’Ivoire.

To this end, two diagnostic tests for intestinal schistosomiasis were used. These were the Kato Katz test, which aims to detect and quantify *S. mansoni* eggs excreted in the faeces, and the POC-CCA test, which detects and quantifies circulating *S. mansoni* antigen.

The KK test is used routinely by schistosomiasis control programmes. According to [40] this test offers many advantages, including high specificity and simultaneous detection of intestinal worm infections, while providing a simple technique that requires minimal supplies and equipment. However, the test offers low sensitivity, particularly in low-intensity infections [11]. It is laborious and can expose technicians to infectious stools [25].

The POC-CCA test is a sensitive and accurate screening tool for *S. mansoni* even in low prevalence areas and uses urine instead of faeces [17, 55]. The main advantages of this test include its easy handling in field conditions and minimal hands-on training requirements for its application [19]. In addition, the intensity of the positive test band allows an estimate of the intensity of infection in infected individuals [16, 48, 54]. However, it has already been shown that the POC-CCA test can overestimate the prevalence of infection due to false positives, particularly in areas of low and moderate prevalence [21, 27] and that its specificity is also influenced by cross-reactions with other helminth infections [44]. According to [68], a single Kato Katz test is 5% cheaper than a single POC-CCA test but the more accurate triple KK test costs 2.4 times as much as a POC-CCA. However, the slightly higher cost of the POC-CCA may be justified if it provides a more sensitive method for detecting schistosomiasis or if this test is more acceptable to participants and practitioners than the stool sampling required to perform the Kato Katz [68].

The two diagnostic methods used in this study gave very different results for the prevalence of *S. mansoni* in the different localities. The POC-CCA test gave higher prevalences of up to 100%, while the highest prevalence detected by KK was 43%. The KK is known to have low sensitivity in low intensity infections and the gold standard for diagnostics is for two tests on each stool for three days with different stool samples, however the increased cost and effort involved means that this is rarely done. The high prevalences obtained by the POC-CCA compared with the KK could be due missed low intensity infections when eggs are not always present in each stool sample but also to the stage of development of the antigen detected by each of the two tests. In the case of schistosomiasis, immature worms during the acute phase or in cases of recent reinfection can produce worm antigens (e.g. CCA) before eggs are excreted in the faeces [26]. This process can give a positive result with the POC-CCA test and a negative result with the Kato-Katz technique [42]. In addition, the difference in prevalence could result from the risk of overestimating prevalence with the POC-CCA [12]. In fact, certain factors such as urinary tract infections, haematuria, the use of diuretics, etc. can have an impact on the positivity of the POC-CCA test, particularly in individuals who are less infected and therefore do not excrete eggs [26, 27]. Some batches of the POC-CCA test (in particular batch 180907091) have been found to give implausible numbers of trace readings which were clearly false positive [22]). We used batch number 190411032 which is (not) known to have this problem.

These facts could be the origin of the large discrepancy between the KK and the POC-CCA when comparing the performances of these two tests, whether the traces are considered positive or not. Several studies have also reported discrepancies between these two tests [13, 48, 61].

There was a lack of significant associations with well-known risk factors in the logistic regression when all the POC-CCA results were used but when the analyses were performed using only the highest intensity infections POC-CCA (+3), these same factors were significantly associated with the probability of *S. mansoni* infection (Table 2). This could be a consequence of low intensity infections being false positives with the POC-CCA but also a consequence of a non-linear relationship between risk factors and infection intensity. This could occur if low intensity infections were a consequence of more effective parasite control by the immune system or immune related suppression of egg secretion as well as or instead of reduced exposure [21]. This is probably why, when the trace was considered positive in this study (Table 5), there was a reduction in specificity and PPV and when the trace was considered negative, there was an increase in specificity when KK was considered the standard. These data are similar to those of [12] who conducted their study in a highly endemic area of north-eastern Brazil. The Schistosomiasis Consortium for Operational Research and Evaluation (SCORE) recommends that trace results should be treated as positives in all places where KK prevalence exceeds a few percent and consequently that the great majority of trace readings in this study should be considered true positives [21].

The very high sensitivity of the POC-CCA in this study makes it somewhat difficult to assess differences in the severity of schistosomiasis. For example, in the localities of Zéaglo and Sahibly, which both have a CCA prevalence of 100%, and a KK prevalence of 9% and 43% respectively on the one hand, and on the other hand in the localities of Pona (27%) and Francdougou (78%) for which CCA were detected but not *S. mansoni* eggs in the subjects, these cases seem difficult to elucidate on the basis of test performance alone. Apart from the fact that a less sensitive methodology would make it easier to differentiate between the two situations and choose the most appropriate intervention, other parameters such as environmental and genetic factors could help to better understand the variability in infection rates specific to this area.

The high prevalence of *S. mansoni* CCA antigen reported in this study indicates high levels of human-parasite contact in the areas concerned and the accessibility of infected human-water contact points. However, the levels determined by the POC-CCA method in the present study are higher than those detected by the same method in recent studies in the same region [8, 10]. Similarly, similar prevalences determined by Kato Katz have also been reported in previous studies conducted in this region [6, 8, 9].

The sex ratio showed a balanced recruitment of girls and boys included in this study. The results of the two tests (POC-CCA and KK) showed that *S. mansoni* was significantly more frequent in boys than in girls, and in schoolchildren aged between 12 and 14. This could be explained by the more frequent contact of older boys with water during their recreational and domestic activities. Several studies have also shown a high prevalence in older boys and schoolchildren [41, 50, 51]. However, contradictory results have been reported by [28, 31, 49] who found no association between infection and gender, and [52] who found a more significant association in girls. These data suggest that behavioural factors influence whether boys or girls are more at risk and that any genetic basis for a higher risk for one sex is weak or non-existent.

Furthermore, this study showed a significant association between activities involving contact with fresh water (swimming, fishing, washing clothes and dishes) and people who secreted *S. mansoni* eggs, those with a +3 infection for the POC-CCA test (Table 2). These results corroborate those of several studies that have revealed the impact of household and recreational activities on *S. mansoni* infection [6, 30, 46, 56]. The majority (61% for KK) of positive schoolchildren had moderate to high intensity of infection. This reflects the high parasite pressure on the population, but also the contamination of the environment, in this case the surface water in the area. These results differ from those of previous studies conducted in the same region [8, 57].

The schools of Sahibly, Yoya and Zéaglo in the departments of Toulepleu and Bloléquin in the Cavally region recorded a high proportion of children with a high intensity of infection. On the other hand, the school in Francdougou, also in the department of Bloléquin in the Cavally region, recorded only one individual with a “+3” infection and no child in this neighbourhood secreted *S. mansoni* eggs. So, although infection pressure is high in the department of Bloléquin and the Cavally region, it is not uniform across the area. This result is confirmed by the spatial distribution results. The schools in Sahibly, Yoya and Zéaglo are the most endemic, which is also the case in Bloléquin, Toulepleu and the Cavally region, at departmental and regional level respectively. This could be due to the proximity of transmission points and the more favourable environmental conditions in these localities compared with others. At Sahibly, a branch of the Cavally river runs through the village and is used for fishing, swimming and other domestic activities. This branch of the river has been the site of hydrographic infrastructures (a bridge) in addition to the gold panning activity carried out on this branch upstream from the village. These environmental conditions are conducive to the proliferation of the vector *Biomphalaria pfeifferi* and its contamination by *S. mansoni*. Previous studies in other countries have established a close relationship between changes in the river environment, the proliferation of the vector and the high rate of human contamination [18, 43, 69].

The results of the intensity of infection analysis based on NB-GLM and NBLH showed a significant association with sex, age, school, department, region, swimming, fishing and washing. Boys had the highest intensity of infection. With regard to the age factor, the intensity of infection increased with age, but children in the 12–14 years age group were the most likely to develop a high-intensity infection. Reinfections in this age group and in boys could be responsible for the high intensities recorded [41, 59]. Region was not significantly associated with intensity of infection, although the department of Toulepleu and the school of Sahibly (Cavally region) were more exposed compared with other departments and schools. Nonetheless, the departments of Guiglo and Bloléquin and certain schools (Francdougou, Golou) also in the Cavally region were less exposed to high intensities of infection, unlike Duékoué (Guémon region) which was more exposed. However, with NB-GLMM, only age and sex remained significantly associated with intensity of infection when the random effect of study sites was included in the model. This observation is probably due to the fact that the site variable is correlated with the environmental variables and therefore masks the effect of the latter. Age and gender, which were not correlated with the site, remained significant and were important risk factors for infection in all study areas. The best predictors of morbidity have been shown to be related to the intensity of infection [23, 34, 45] and efforts to reduce the impact of these environmental risk factors should therefore continue.

## Conclusion

Accurate and practical methods are essential for reliable field diagnosis of schistosomiasis, monitoring treatment success, and evaluating control programmes. This study examined the epidemiological situation of intestinal schistosomiasis using two diagnostic methods in the endemic zone of western Côte d’Ivoire. The results obtained in this study show that the POC-CCA test is a valuable method that is easy to use and accurate in a schistosomiasis context. Nevertheless, the KK test has clear advantages. It is much more specific than the POC-CCA test and at the same time other helminths can be detected [39]. However, it would be useful to combine the two techniques in epidemiological investigations. This would increase the likelihood of detecting individuals with low levels of infection intensity in endemic areas and allow accurate measurement of disease burden when planning intervention measures at the community level. In addition, this study shows that the prevalence is high and infection intensities generally range from moderate to high. The study area has heterogeneous *S. mansoni* infection pressure, and the most important local epidemiological factors are sex and age. Awareness campaigns, health education, provision of safe drinking water, increased use of latrines, control of snails and deworming of schoolchildren are essential and should be proportional to the parasite pressure.

## References

[1] Abou-El-Naga IF. 2018. Towards elimination of schistosomiasis after 5000 years of endemicity in Egypt. Acta Tropica, 181, 112–121. 29453950 10.1016/j.actatropica.2018.02.005

[2] Adenowo AF, Oyinloye BE, Ogunyinka BI, Kappo AP. 2015. Impact of human schistosomiasis in sub-Saharan Africa. Brazilian Journal of Infectious Diseases, 19(2), 196–205. 10.1016/j.bjid.2014.11.004 PMC9425372 25636189

[3] Adoubryn KD, Ouhon J, Yapo CG, Assoumou EY, Ago KML, Assoumou A. 2006. Epidemiological profile of the schistosomiasis in school children in the Agneby Region (south-east of Côte-d’Ivoire). Bulletin de la Société de Pathologie Exotique, 99(1), 28–31. 16568679 10.3185/pathexo2644

[4] Adoubryn KD, Kouadio-Yapo CG, Ouhon J, Aka N, Bintto F, Assoumou A. 2012. Parasitoses intestinales infantiles à Biankouma, région des 18 Montagnes (ouest de la Côte d’Ivoire): étude de l’efficacité et de la tolérance du praziquantel et de l’albendazole. Médecine et Santé Tropicales, 22(2), 170–176. 23107664 10.1684/mst.2012.0048

[5] Amuta E, Houmsou R. 2014. Prevalence, intensity of infection and risk factors of urinary schistosomiasis in pre-school and school aged children in Guma Local Government Area, Nigeria. Asian Pacific Journal of Tropical Medicine, 7(1), 34–39. 24418080 10.1016/S1995-7645(13)60188-1

[6] Angora EK, Boissier J, Menan H, Rey O, Tuo K, Touré OA, Coulibaly JT, Méité A, Raso G, N’Goran EK, Jürg Utzinger J, Oliver Balmer O. 2019. Prevalence and risk factors for schistosomiasis among schoolchildren in two settings of Côte d’Ivoire. Tropical Medicine and Infectious Disease, 4(3), 110. 31340504 10.3390/tropicalmed4030110 PMC6789509

[7] Assaré RK, Lai YS, Yapi A, Tian-Bi YNT, Ouattara M, Yao PK, Knopp S, Vounatsou P, Utzinger J, N’Goran EK. 2015. The spatial distribution of *Schistosoma mansoni* infection in four regions of western Côte d’Ivoire. Geospatial Health, 10(1), 345. 26054523 10.4081/gh.2015.345

[8] Assaré RK, N’Tamon RN, Bellai LG, Koffi JA, Mathieu TBI, Ouattara M, Eveline Hürlimann E, Coulibaly JT, Diabaté S, N’Goran EK, Utzinger J. 2020. Characteristics of persistent hotspots of *Schistosoma mansoni* in western Côte d’Ivoire. Parasites & Vectors, 13, 337. 32616074 10.1186/s13071-020-04188-x PMC7333430

[9] Assaré RK, Tian-Bi YNT, Yao PK, N’Guessan NA, Ouattara M, Yapi A, Coulibaly JT, Meïté A, Hürlimann E, Stefanie Knopp S, Utzinger J, N’Goran EK. 2016. Sustaining control of schistosomiasis mansoni in Western Côte d’Ivoire: results from a SCORE Study, one year after initial praziquantel administration. PLoS Neglected Tropical Diseases, 10(1), e0004329. 26789749 10.1371/journal.pntd.0004329 PMC4720284

[10] Assaré RK, Tra MBI, Ouattara M, Hürlimann E, Coulibaly JT, N’Goran EK, Utzinger J. 2018. Sensitivity of the point-of-care circulating cathodic antigen urine cassette test for diagnosis of *Schistosoma mansoni* in low-endemicity settings in Côte d’Ivoire. American Journal of Tropical Medicine and Hygiene, 99(6), 1567–1572. 30277203 10.4269/ajtmh.18-0550 PMC6283482

[11] Bärenbold O, Garba A, Colley DG, Fleming FM, Haggag AA, Ramzy RMR, Assaré RK, Tukahebwa EM, Mbonigaba JB, Bucumi V, Kebede B, Yibi MS, Meité A, Coulibaly JT, N’Goran EK, Tchuem Tchuenté LA, Mwinzi P, Utzinger J, Vounatsou P. 2018. Translating preventive chemotherapy prevalence thresholds for *Schistosoma mansoni* from the Kato-Katz technique into the point-of-care circulating cathodic antigen diagnostic test. PLoS Neglected Tropical Diseases, 12(12), e0006941. 30550594 10.1371/journal.pntd.0006941 PMC6310297

[12] Bezerra DF, Pinheiro MCC, Barbosa L, Viana AG, Fujiwara RT, Bezerra FSM. 2021. Diagnostic comparison of stool exam and point-of-care circulating cathodic antigen (POC-CCA) test for *Schistosomiasis mansoni* diagnosis in a high endemicity area in northeastern Brazil. Parasitology, 148(4), 420–426. 33190646 10.1017/S0031182020002164 PMC11010182

[13] Bezerra FSM, Leal JKF, Sousa MS, Pinheiro MCC, Ramos AN Jr, Silva-Moraes V, Katz N. 2018. Evaluating a point-of-care circulating cathodic antigen test (POC-CCA) to detect *Schistosoma mansoni* infections in a low endemic area in north-eastern Brazil. Acta Tropica, 182, 264–270. 29526480 10.1016/j.actatropica.2018.03.002

[14] Bergquist R, Johansen MV, Utzinger J. 2009. Diagnostic dilemmas in helminthology: what tools to use and when? Trends in Parasitology, 25(4), 151–156. 19269899 10.1016/j.pt.2009.01.004

[15] Calasans TAS, Souza GTR, Melo CM, Madi RR, Jeraldo VDLS. 2018. Socioenvironmental factors associated with *Schistosoma mansoni* infection and intermediate hosts in an urban area of northeastern Brazil. PLoS One, 13(5), e0195519. 29718924 10.1371/journal.pone.0195519 PMC5931446

[16] Casacuberta-Partal M, Hoekstra PT, Kornelis D, van Lieshout L, van Dam GJ. 2019. An innovative and user-friendly scoring system for standardised quantitative interpretation of the urine-based point-of-care strip test (POC-CCA) for the diagnosis of intestinal schistosomiasis: a proof-of-concept study. Acta Tropica, 199, 105150. 31425672 10.1016/j.actatropica.2019.105150

[17] Clements MN, Corstjens PLAM, Binder S, Campbell CH Jr, de Dood CJ, Fenwick A, Harrison W, Kayugi D, King CH, Kornelis D, Ndayishimiye O, Ortu G, Lamine MS, Zivieri A, Colley DG, van Dam GJ. 2018. Latent class analysis to evaluate performance of point-of-care CCA for low-intensity *Schistosoma mansoni* infections in Burundi. Parasites & Vectors, 11(1), 111. 29475457 10.1186/s13071-018-2700-4 PMC5824563

[18] Coelho PRS, Ker FTO, Araújo AD, RicardoJPS Guimarães, Negrão-Corrêa DA, Caldeira RL, Geiger SM. 2021. Identification of risk areas for intestinal schistosomiasis, based on malacological and environmental data and on reported human cases. Frontiers in Medicine, 8, 642348. 34422845 10.3389/fmed.2021.642348 PMC8377395

[19] Colley DG, Andros TS, Campbell CH. 2017. Schistosomiasis is more prevalent than previously thought: what does it mean for public health goals, policies, strategies, guidelines and intervention programs? Infectious Diseases of Poverty, 6(1), 63. 28327187 10.1186/s40249-017-0275-5 PMC5361841

[20] Colley DG, Bustinduy AL, Secor WE, King CH. 2014. Human schistosomiasis. Lancet, 383(9936), 2253–2264. 24698483 10.1016/S0140-6736(13)61949-2 PMC4672382

[21] Colley DG, King CH, Kittur N, Ramzy RMR, Secor WE, Fredericks-James M, Ortu G, Clements MN, Ruberanziza E, Umulisa I, Wittmann U, Campbell CH. 2020. Evaluation, validation, and recognition of the point-of-care circulating cathodic antigen, urine-based assay for mapping *Schistosoma mansoni* infections. American Journal of Tropical Medicine and Hygiene, 103(1_Suppl), 42–49. 32400347 10.4269/ajtmh.19-0788 PMC7351311

[22] Colley DG, Ramzy RMR, Maganga J, Kinung’hi S, Odiere MR, Musuva RM, Campbell CH Jr. 2023. The POC-CCA assay for detection of schistosoma mansoni infection needs standardization in production and proper quality control to be reliable. Acta Tropica, 238, 106795. 36539024 10.1016/j.actatropica.2022.106795

[23] Davis A. 2004. Clinical trials in parasitic diseases. Transactions of the Royal Society of Tropical Medicine and Hygiene, 98(3), 139–141. 15024922 10.1016/s0035-9203(03)00036-1

[24] Elmorshedy H, Bergquist R, Fayed A, Guirguis W, Abdel-Gawwad E, Eissa S, Barakat R. 2020. Elimination of schistosomiasis requires multifactorial diagnostics: evidence from high- and low-prevalence areas in the Nile Delta, Egypt. Infectious Diseases of Poverty, 9(1), 31. 32241298 10.1186/s40249-020-00648-9 PMC7119160

[25] Enk MJ, Lima AC, Drummond SC, Schall VT, Coelho PM. 2008. The effect of the number of stool samples on the observed prevalence and the infection intensity with *Schistosoma mansoni* among a population in an area of low transmission. Acta Tropica, 108(2–3), 222–228. 18973744 10.1016/j.actatropica.2008.09.016

[26] Ferreira FT, Fidelis TA, Pereira TA, Otoni A, Queiroz LC, Amâncio FF, Antunes CM, Lambertucci JR. 2017. Sensitivity and specificity of the circulating cathodic antigen rapid urine test in the diagnosis of *Schistosomiasis mansoni* infection and evaluation of morbidity in a low- endemic area in Brazil. Revista da Sociedade Brasileira de Medicina Tropical, 50(3), 358–364. 28700054 10.1590/0037-8682-0423-2016

[27] Fuss A, Mazigo HD, Tappe D, Kasang C, Mueller A. 2018. Comparison of sensitivity and specificity of three diagnostic tests to detect *Schistosoma mansoni* infections in school children in Mwanza region, Tanzania. PLoS One, 13(8), e0202499. 30133490 10.1371/journal.pone.0202499 PMC6105001

[28] Gazzinelli A, Velasquez-Melendez G, Crawford SB, LoVerde PT, Correa-Oliveira R, Kloos H. 2006. Socioeconomic determinants of schistosomiasis in a poor rural area in Brazil. Acta Tropica, 99(2–3), 260–271. 17045559 10.1016/j.actatropica.2006.09.001 PMC1828742

[29] Grenfell R, Harn DA, Tundup S, Da’dara A, Siqueira L, Coelho PMZ. 2013. New approaches with different types of circulating cathodic antigen for the diagnosis of patients with low *Schistosoma mansoni* load. PLoS Neglected Tropical Diseases, 7(2), e2054. 23469295 10.1371/journal.pntd.0002054 PMC3585039

[30] Hailu T, Mulu W, Abera B. 2020. Effects of water source, sanitation and hygiene on the prevalence of *Schistosoma mansoni* among school age children in Jawe District, Northwest Ethiopia. Iranian Journal of Parasitology, 15(1), 124–129. 32489384 PMC7244839

[31] Hajissa K, Muhajir AEMA, Eshag HA, Alfadel A, Nahied E, Dahab R, Ali SM, Mohammed M, Gaafar M, Mohamed Z. 2018. Prevalence of schistosomiasis and associated risk factors among school children in Um-Asher Area, Khartoum, Sudan. BMC Research Notes, 11, 779. 30382901 10.1186/s13104-018-3871-y PMC6211415

[32] Hinz R, Schwarz NG, Hahn A, Frickmann H. 2017. Serological approaches for the diagnosis of schistosomiasis – a review. Molecular and Cellular Probes, 31, 2–21. 27986555 10.1016/j.mcp.2016.12.003

[33] Hoekstra PT, Casacuberta-Partal M, van Lieshout L, Corstjens PLAM, Tsonaka R, Assaré RK, Silué KD, Meité A, N’Goran EK, N’Gbesso YK, Amoah AS, Roestenberg M, Knopp S, Utzinger J, Coulibaly JT, van Dam GJ. 2020. Efficacy of single versus four repeated doses of praziquantel against *Schistosoma mansoni* infection in school-aged children from Côte d’Ivoire based on Kato-Katz and POC-CCA: an open-label, randomised controlled trial (RePST. PLoS Neglected Tropical Diseases, 14(3), e0008189. 32196506 10.1371/journal.pntd.0008189 PMC7112237

[34] Ismail HAHA, Hong ST, Babiker ATEB, Hassan RMAE, Sulaiman MAZ, Jeong HG, Kong WH, Lee SH, Cho HI, Nam HS, Oh CH, Lee YH. 2014. Prevalence, risk factors, and clinical manifestations of schistosomiasis among school children in the White Nile River basin, Sudan. Parasites & Vectors, 7, 478. 25312470 10.1186/s13071-014-0478-6 PMC4200116

[35] Joof E, Sanyang AM, Camara Y, Sey AP, Baldeh I, Jah SL, Ceesay SJ, Sambou SM, Sanyang S, Wade CM, Sanneh B. 2021. Prevalence and risk factors of schistosomiasis among primary school children in four selected regions of The Gambia. PLoS Neglected Tropical Diseases, 15(5), e0009380. 33974623 10.1371/journal.pntd.0009380 PMC8139473

[36] Jones IJ, Sokolow SH, Chamberlin AJ, Lund AJ, Jouanard N, Bandagny L, Ndione R, Senghor S, Schacht AM, Riveau G, Hopkins SR, Rohr JR, Remais JV, Lafferty KD, Kuris AM, Wood CL, De Leo G. 2021. Schistosome infection in Senegal is associated with different spatial extents of risk and ecological drivers for *Schistosoma haematobium* and *S. mansoni*. PLoS Neglected Tropical Diseases, 15(9), e0009712. 34570777 10.1371/journal.pntd.0009712 PMC8476036

[37] Kalinda C, Chimbari M, Mukaratirwa S. 2017. Implications of changing temperatures on the growth, fecundity and survival of intermediate host snails of schistosomiasis: a systematic review. International Journal of Environmental Research and Public Health, 14(1), 80. 28098789 10.3390/ijerph14010080 PMC5295331

[38] Katz N, Chaves A, Pellegrino J. 1972. A simple device for quantitative stool thick-smear technique in schistosomiasis mansoni. Revista do Instituto de Medicina Tropical de São Paulo, 14(6), 397–400. 4675644

[39] King CH. 2010. Parasites and poverty: the case of schistosomiasis. Acta Tropica, 113(2), 95–104. 19962954 10.1016/j.actatropica.2009.11.012 PMC2812649

[40] Kongs A, Marks G, Verlé P, Van der Stuyft P. 2001. The unreliability of the Kato-Katz technique limits its usefulness for evaluating *S. mansoni* infections. Tropical Medicine & International Health, 6(3), 163–169. 11299032 10.1046/j.1365-3156.2001.00687.x

[41] Lamberti O, Kabatereine NB, Tukahebwa EM, Chami GF. 2021. *Schistosoma mansoni* infection risk for school-aged children clusters within households and is modified by distance to freshwater bodies. PLoS One, 16(11), e0258915. 34735487 10.1371/journal.pone.0258915 PMC8568121

[42] Lambertucci JR, Drummond SC, Voieta I, de Queiróz LC, Pereira PP, Chaves BA, Botelho PP, Prata PH, Otoni A, Vilela JF, Antunes CM. 2013. An outbreak of acute *Schistosoma mansoni* schistosomiasis in a nonendemic area of Brazil: a report on 50 cases, including 5 with severe clinical manifestations. Clinical Infectious Diseases, 57(1), e1–e6. 23532472 10.1093/cid/cit157

[43] Li YS, Raso G, Zhao ZY, He YK, Ellis MK, McManus DP. 2007. Large water management projects and schistosomiasis control, Dongting Lake Region, China. Emerging Infectious Diseases, 13(7), 973–979. 18214167 10.3201/eid1307.070848 PMC2878251

[44] Lodh N, Mwansa JC, Mutengo MM, Shiff CJ. 2013. Diagnosis of *Schistosoma mansoni* without the stool: comparison of three diagnostic tests to detect *Schistosoma* [corrected] *mansoni* infection from filtered urine in Zambia. American Journal of Tropical Medicine and Hygiene, 89(1), 46–50. 23716406 10.4269/ajtmh.13-0104 PMC3748486

[45] Mawa PA, Kincaid-Smith J, Tukahebwa EM, Webster JP, Wilson S. 2021. Schistosomiasis morbidity hotspots: roles of the human host, the parasite and their interface in the development of severe morbidity. Frontiers in Immunology, 12, 635869. 33790908 10.3389/fimmu.2021.635869 PMC8005546

[46] M’Bra RK, Kone B, Yapi YG, Silué KD, Sy I, Vienneau D, Soro N, Cissé G, Utzinger J. 2018. Risk factors for schistosomiasis in an urban area in northern Côte d’Ivoire. Infectious Diseases of Poverty, 7(1), 47. 29773076 10.1186/s40249-018-0431-6 PMC5958400

[47] Menjetta T, Debalke S, Dana D. 2019. *Schistosoma mansoni* infection and risk factors among the fishermen of Lake Hawassa, southern Ethiopia. Journal of Biosocial Science, 51(6), 817–826. 30838967 10.1017/S0021932019000075

[48] Mewamba EM, Tiofack AAZ, Kamdem CN, Ngassam RIK, Mbagnia MCT, Nyangiri O, Noyes H, Womeni HM, Njiokou F, Simo G. 2021. Field assessment in Cameroon of a reader of POC-CCA lateral flow strips for the quantification of *Schistosoma mansoni* circulating cathodic antigen in urine. PLoS Neglected Tropical Diseases, 15(7), e0009569. 34260610 10.1371/journal.pntd.0009569 PMC8312929

[49] Mewamba EM, Tiofack AAZ, Kamdem CN, Tchounkeu EY, Tatang RJA, Mengoue LET, Mbagnia MCT, Njiokou F, Casacuberta-Partal M, Womeni HM, Simo G, TrypanoGEN+ Research Group of the H3Africa Consortium. 2022. Fine-scale mapping of *Schistosoma mansoni* infections and infection intensities in sub-districts of Makenene in the Centre region of Cameroon. PLoS Neglected Tropical Diseases, 16(10), e0010852. 36227962 10.1371/journal.pntd.0010852 PMC9595529

[50] Mnkugwe RH, Minzi OS, Kinung’hi SM, Kamuhabwa AA, Aklillu E. 2020. Prevalence and correlates of intestinal schistosomiasis infection among school-aged children in North-Western Tanzania. PLoS One, 15(2), e0228770. 32023307 10.1371/journal.pone.0228770 PMC7001966

[51] Mueller A, Fuss A, Ziegler U, Kaatano GM, Mazigo HD. 2019. Intestinal schistosomiasis of Ijinga Island, north-western Tanzania: prevalence, intensity of infection, hepatosplenic morbidities and their associated factors. BMC Infectious Diseases, 19(1), 832. 31590657 10.1186/s12879-019-4451-z PMC6781372

[52] Ndamukong KJ, Ayuk MA, Dinga JS, Akenji TN, Ndiforchu VA, Titanji VP. 2000. The pattern of soil-transmitted nematode infections in primary school children of the Kumba Health District, South-West Cameroon. African Journal of Health Sciences, 7(3–4), 103–106. 17650034

[53] N’gbesso NJ-P, N’guessan NA, Assaré RK, Orsot MN, N’dri K, Yapi A. 2017. Epidemiology of schistosomiasis in Ahoué, southern Côte d’Ivoire. International Journal of Innovation and Applied Studies, 21(3), 378–387.

[54] Nyangiri OA, Edwige SA, Koffi M, Mewamba E, Simo G, Namulondo J, Mulindwa J, Nassuuna J, Elliott A, Karume K, Mumba D, Corstjens PLAM, Casacuberta-Partal M, van Dam GJ, Bucheton B, Noyes H, Matovu E, TrypanoGEN+ Research Group of the H3Africa Consortium. 2021. Candidate gene family-based and case-control studies of susceptibility to high *Schistosoma mansoni* worm burden in African children: a protocol. AAS Open Research, 4, 36. 35252746 10.12688/aasopenres.13203.1 PMC8861467

[55] Okoyo C, Simiyu E, Njenga SM, Mwandawiro C. 2018. Comparing the performance of circulating cathodic antigen and Kato-Katz techniques in evaluating *Schistosoma mansoni* infection in areas with low prevalence in selected counties of Kenya: a cross-sectional study. BMC Public Health, 18(1), 478. 29642875 10.1186/s12889-018-5414-9 PMC5896080

[56] Onyekwere AM, Rey O, Nwanchor MC, Alo M, Angora EK, Allienne JF, Boissier J. 2022. Prevalence and risk factors associated with urogenital schistosomiasis among primary school pupils in Nigeria. Parasite Epidemiology and Control, 18, e00255. 35832869 10.1016/j.parepi.2022.e00255 PMC9272031

[57] Ouattara M, Diakité NR, Yao PK, Saric J, Coulibaly JT, Assaré RK, Bassa FK, Koné N, Guindo-Coulibaly N, Hattendorf J, Utzinger J, N’Goran EK. 2021. Effectiveness of school-based preventive chemotherapy strategies for sustaining the control of schistosomiasis in Côte d’Ivoire: results of a 5-year cluster randomized trial. PLoS Neglected Tropical Diseases, 15(1), e0008845. 33449924 10.1371/journal.pntd.0008845 PMC7810315

[58] Pennington L, Hsieh M. 2014. The immunobiology of urogenital schistosomiasis in Immune response to parasitic infections, in Bentham science, vol. 2, Jirillo E, Magrone T, Miragliota G, Editors. p. 93–124.

[59] Pinot de Moira A, Fulford AJ, Kabatereine NB, Ouma JH, Booth M, Dunne DW. 2010. Analysis of complex patterns of human exposure and immunity to *Schistosomiasis mansoni*: the influence of age, sex, ethnicity and IgE. PLoS Neglected Tropical Diseases, 4(9), e820. 20856909 10.1371/journal.pntd.0000820 PMC2939029

[60] R Core Team. 2024. _R: A Language and Environment for Statistical Computing_. R Foundation for Statistical Computing, Vienna, Austria. <https://www.R-project.org/>.

[61] Sassa M, Chadeka EA, Cheruiyot NB, Tanaka M, Moriyasu T, Kaneko S, Njenga SM, Cox SE, Hamano S. 2020. Prevalence and risk factors of *Schistosoma mansoni* infection among children under two years of age in Mbita, Western Kenya. PLoS Neglected Tropical Diseases, 14(8), e0008473. 32841228 10.1371/journal.pntd.0008473 PMC7447014

[62] Sokouri EA, Ahouty Ahouty B, N’Djetchi M, Abé IA, Yao BGFD, Konan TK, MacLeod A, Noyes H, Nyangiri O, Matovu E, Koffi M, TrypanoGEN+ Research Group of the H3Africa Consortium. 2024. Impact of environmental factors on *Biomphalaria pfeifferi* vector capacity leading to human infection by *Schistosoma mansoni* in two regions of western Côte d’Ivoire. Parasites & Vectors, 17(1), 179. 38581062 10.1186/s13071-024-06163-2 PMC10996162

[63] Vale N, Gouveia MJ, Rinaldi G, Brindley PJ, Gärtner F, Correia da Costa JM. 2017. Praziquantel for schistosomiasis: single-drug metabolism revisited, mode of action, and resistance. Antimicrobial Agents and Chemotherapy, 61(5), e02582-16. 28264841 10.1128/AAC.02582-16 PMC5404606

[64] World Health Organization. 2011. Increasing access to diagnostics through technology transfer and local production. World Health Organization: Geneva.

[65] World Health Organization. 2020. Current estimated total number of individuals with morbidity and mortality due to *Schistosomiasis haematobium* and *S. mansoni* infection in Sub-saharan Africa. https://www.who.int/teams/control-of-neglected-tropical-diseases/schistosomiasis/epidemiology.

[66] World Health Organization. 2019. Bench aids for the diagnosis of intestinal parasites, 2nd edn. 32 p. https://www.who.int/publications-detail-redirect/9789241515344.

[67] World Health Organization. 2002. Prevention and control of schistosomiasis and soil-transmitted helminthiasis: report of a WHO expert committee. World Health Organization: Geneva. https://apps.who.in/iris/handle/10665/42588. 12592987

[68] Worrell CM, Bartoces M, Karanja DM, Ochola EA, Matete DO, Mwinzi PN, Montgomery SP, Secor WE. 2015. Cost analysis of tests for the detection of *Schistosoma mansoni* infection in children in western Kenya. American Journal of Tropical Medicine and Hygiene, 92(6), 1233–1239. 25870422 10.4269/ajtmh.14-0644 PMC4457354

[69] Zhou YB, Liang S, Chen Y, Jiang QW. 2016. The Three Gorges Dam: does it accelerate or delay the progress towards eliminating transmission of schistosomiasis in China? Infectious Diseases of Poverty, 5(1), 63. 27377962 10.1186/s40249-016-0156-3 PMC4932735

